# Toward Objective Wound Edge Classification in Clinical Practice

**DOI:** 10.1111/exd.70287

**Published:** 2026-06-05

**Authors:** Corrado Zengarini, Tommaso Giacometti, Yuri Merli, Davide Griffa, Luca Rapparini, Alessio Natale, Michele Fruci, Gastone Castellani, Daniel Remondini, Michelangelo La Placa, Alessandro Pileri, Michela Starace, Nico Curti

**Affiliations:** ^1^ Department of Medical and Surgical Sciences University of Bologna Bologna Italy; ^2^ Dermatology Unit IRCCS Azienda Ospedaliero‐Universitaria Di Bologna Bologna Italy; ^3^ Department of Physics and Astronomy University of Bologna Bologna Italy; ^4^ INFN Bologna Italy; ^5^ Neuroscience Biobank of Bologna (BNB) IRCCS Istituto Delle Scienze Neurologiche Di Bologna Bologna Italy; ^6^ IRCCS Azienda Ospedaliero Universitaria Di Bologna Bologna Italy

**Keywords:** computer vision, machine learning, surface meshing, wound, wound healing

## Abstract

Wound edge assessment is a key component of chronic wound evaluation, but it remains highly subjective and affected by inter‐observer variability, particularly when performed on two‐dimensional clinical photographs. We retrospectively analysed 1 860 wound images acquired during routine clinical practice and independently annotated by four expert clinicians. An automated image‐analysis pipeline was used to segment the wound, standardise the peri‐wound border region, and estimate the three‐dimensional profile of the wound edge. We first tested whether geometry‐derived edge profiles alone could reproduce clinical wound edge categories. We then evaluated whether adding global visual descriptors of wound shape, colour appearance, and surface pattern improved agreement with clinicians. Inter‐clinician agreement was low, confirming the intrinsic subjectivity of wound edge classification. Geometry‐based analysis identified coherent edge‐profile patterns but showed poor correspondence with clinical annotations. In contrast, a supervised classifier incorporating both geometric and visual features achieved agreement comparable to, and in some comparisons higher than, the agreement observed among clinicians. Clinical wound edge assessment is not driven by edge geometry alone. Visual cues such as wound shape, colour appearance, and surface pattern appear to influence expert classification and may contribute to variability. Automated image‐based analysis may support more reproducible wound edge assessment, provided that it is externally validated in diverse clinical settings.

## 1 Introduction

Chronic wounds represent a silent yet pervasive crisis in clinical medicine, affecting millions of people worldwide [1]: these wounds, which arise from various underlying conditions such as diabetes, vascular diseases, or prolonged pressure, deviate from the typical healing trajectory [2, 3], persisting for months or even years. The high prevalence of these wounds, along with associated physical complications such as pain and the severe psychological and social burden [4, 5, 6, 7], underscores their substantial impact on patients. Furthermore, managing chronic wounds places a considerable financial burden on healthcare systems worldwide, accounting for approximately 3% of healthcare expenditures [8, 9, 10]. A significant portion of these costs is associated with professional wound care, including the time spent on healthcare providers and hospitalisations [11, 12, 13].

Despite the crucial importance of accurate wound assessment, traditional methods [14, 15, 16] remain predominantly subjective [15, 17], heavily relying on clinician expertise and visual inspection. Studies indicate that inter‐operator variability in wound evaluation can reach up to 30% during clinical practice [17, 18], even among experienced professionals. A key source of this inconsistency lies in multi‐item scoring systems such as the Bates‐Jensen Wound Assessment Tool (BWAT) [19, 20, 21] and the Photographic Wound Assessment Tool (PWAT) [22, 23], where certain parameters are inherently more subjective than others [24, 25, 26, 27, 28]. Among these, wound edge classification represents one of the most challenging and variable aspects.

Both BWAT and PWAT incorporate edge assessment, but their classification criteria introduce significant room for interpretation. The BWAT categorises wound edges into five distinct types: Indistinct, Distinct, Well‐defined, Rolled‐under, and Fibrotic, while PWAT evaluates edges on a four‐point scale, ranging from completely attached to significantly detached or rolled under. The variability in classifying edge types contributes substantially to discrepancies between clinicians [29, 30, 31], affecting the consistency and reproducibility of wound scoring [23, 32, 33].

To address this unmet need, we developed an automated image‐based framework aimed at quantifying wound edge morphology from routine clinical photographs. The approach focuses primarily on the transition between peri‐wound skin and the wound bed, which represents the anatomical basis of edge assessment in clinical scoring systems.

We first investigated whether simple geometric profiles of the wound edge were sufficient to reproduce expert classifications. We then evaluated whether the inclusion of additional visual information, such as global wound shape, colour appearance, and surface pattern, improved agreement with clinicians. This two‐step strategy allowed us to test whether clinical wound edge classification reflects a shared geometric ground truth or whether it is influenced by broader visual cues not explicitly captured by standard scoring definitions.

## 2 Methods

### 2.1 Study Design and Clinical Image Dataset

This retrospective observational study included 1 860 wound images acquired during routine clinical practice at the Dermatology Unit of IRCCS Sant'Orsola‐Malpighi University Hospital, Bologna, Italy. Images were collected using smartphone cameras without a rigid acquisition protocol to reflect real‐world clinical and telemedicine conditions. All images were stored in 8‐bit RGB format with a resolution of 1 440 × 1 080 pixels. The acquisition protocol and part of the dataset have been previously described. A detailed description of the patient population is provided in the Supporting Information.

Wounds were photographed as part of routine documentation and longitudinal monitoring. When available, clinical images included a centimetre scale and colour calibration reference. No additional clinical information, palpation, alternative viewing angles, or three‐dimensional acquisition was available to the annotators during the image‐based evaluation.

### 2.2 Clinical Wound Edge Annotation and Inter‐Rater Agreement

Each image was independently evaluated by four expert clinicians. Clinicians classified wound edges according to the wound edge categories used in our BWAT/PWAT‐derived clinical assessment workflow, including indistinct, attached, not attached, rolled‐under, hyperkeratotic, and fibrotic edges. The distribution of annotations for each clinician is reported in Table 1.

**TABLE 1:** Expert clinicians' annotation. For each expert clinician, the number of images assigned to each wound edge category used in the local BWAT/PWAT‐derived clinical assessment workflow is reported. percentages refer to the total dataset.

Expert	Indistinct	Attached	Not attached	Rolled‐under	Hyperkeratotic	Fibrotic
e1	57 (3.1%)	718 (38.6%)	523 (28.1%)	83 (4.5%)	255 (13.7%)	224 (12.0%)
e2	115 (6.2%)	612 (39.2%)	788 (42.4%)	207 (11.1%)	72 (3.9%)	66 (3.5%)
e3	84 (4.5%)	981 (52.7%)	231 (12.4%)	82 (4.4%)	400 (21.5%)	82 (4.4%)
e4	67 (3.6%)	893 (48.0%)	634 (34.1%)	90 (4.8%)	149 (8.0%)	27 (1.5%)

Following expert consensus, the rolled‐under and fibrotic categories were merged for subsequent analyses because of their substantial visual overlap and limited reproducibility on two‐dimensional images.

Inter‐rater agreement among clinicians was quantified using Cohen's κ coefficient. Cohen's κ measures categorical agreement beyond chance and is more appropriate than raw accuracy in the presence of class imbalance. Values range from −1 to 1, with 0 indicating chance‐level agreement and 1 indicating perfect agreement.

### 2.3 Automated Wound Segmentation, Depth Estimation, and Edge Standardisation

To enable quantitative analysis of the wound edge, each image was processed using an automated image‐analysis pipeline. First, the wound area was segmented to identify the wound contour and separate the lesion from the surrounding skin and background. Only the largest wound component was retained for subsequent analyses, to avoid secondary lesions orartefacts. Automated segmentations were visually checked by expert clinicians.

Second, a depth map was estimated from each two‐dimensional photograph to obtain an approximate representation of the three‐dimensional wound surface. The wound mask and the corresponding depth map were then combined to characterise the transition between peri‐wound skin and the wound bed.

**FIGURE 1:**
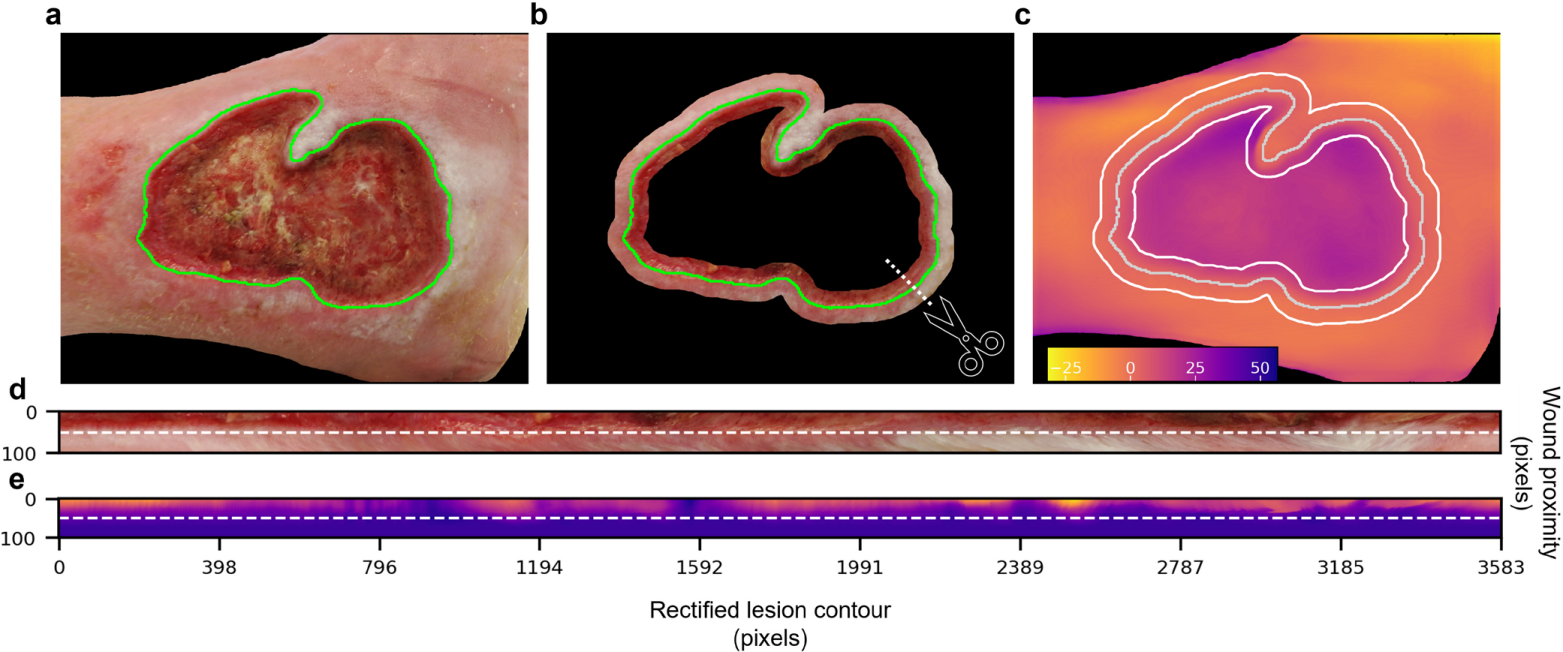
Image rectification procedure. Schematic representation of the pre‐processing pipeline applied on the wound images: (a) raw image with highlighted wound edges identified; (b) extraction of the wound transition area obtained by applying a series of erosions and dilations on the wound mask; (c) depth‐map on which are reported the corresponding wound and wound transition contours; (d, e) rectified wound transition areas estimated on both the raw and depth‐map images; the dashed line highlights the transition between the inner and outer part of the wound (border), according to the identified lesion edge; the reported depth‐map is processed applying a linear fit to mitigate artefacts due to wound orientations.

Because wound contours are irregular and image acquisition conditions are heterogeneous, the peri‐wound transition region was geometrically standardised before feature extraction. This rectification procedure transformed the irregular wound border into a common spatial reference, allowing the edge profile to be analysed consistently across images. The resulting standardised edge region was used to extract depth profiles from the outside of the wound toward the wound bed (Figure 1).

Full technical details of the segmentation model (*Deepskin* [23, 34]), depth‐estimation model [35] (*Depth‐Anything‐V2* [36]), and rectification algorithm are provided in the Supporting Information.

### 2.4 Feature Extraction: Geometric and Visual Wound Descriptors

Two groups of image‐derived descriptors were extracted. The first group included geometric descriptors directly related to the wound edge. These features described the size and shape of the wound, the regularity of the border, and the depth profile of the transition between peri‐wound skin and wound bed. These descriptors were considered the closest computational approximation of the clinical concept of wound edge morphology.

The second group included additional visual descriptors describing the global appearance of the wound and peri‐wound region, including chromatic appearance and surface pattern. These descriptors were not considered part of the strict geometric definition of the wound edge. They were included to test whether clinicians may implicitly rely on visual information beyond edge geometry when assigning wound edge categories.

The complete list of extracted features and their computational definitions is reported in the Supporting Information.

### 2.5 Unsupervised Geometry‐Based Analysis of Wound Edge Profiles

We first performed an unsupervised analysis to test whether wound edge geometry alone was sufficient to define clinically meaningful wound edge groups. In this analysis, clinical annotations were not used to create the groups. Instead, wounds were grouped only according to the similarity of their depth‐derived edge profiles.

Before grouping, each edge profile was summarised according to simple geometric trends representing different transitions between peri‐wound skin and wound bed, including flat, linear, abrupt, or smooth transitions. These trends were designed to provide an intuitive geometric counterpart to clinical wound edge categories.

After the unsupervised groups had been generated, they were compared post hoc with clinicians' annotations. This comparison was used to determine whether geometry‐derived groups corresponded to the wound edge categories assigned in clinical practice. Agreement between unsupervised groups and clinical labels was quantified using chance‐corrected agreement metrics.

The mathematical functions used to summarise edge profiles, the dimensionality‐reduction method [37], clustering procedure, label‐alignment strategy, and agreement metrics are reported in the Supporting Information.

### 2.6 Supervised Analysis: Reproducing Clinicians' Wound Edge Classifications

We then performed a supervised classification analysis to evaluate whether clinicians' wound edge labels could be reproduced from image‐derived features. In contrast to the unsupervised analysis, clinical annotations were used during model training.

Two supervised models were compared. The first model used only depth‐derived edge‐profile features and was designed to test whether edge geometry alone could reproduce clinical classifications. The second model used the full set of extracted descriptors, including edge geometry, global wound shape, chromatic appearance, and surface pattern. This comparison allowed us to evaluate whether non‐geometric visual information improved agreement with clinicians.

Because each image was independently annotated by four clinicians, model performance was evaluated against each clinician separately. The resulting model‐clinician agreement was then compared with the agreement observed among clinicians. This design allowed us to assess whether the supervised model captured a shared component of clinical judgement and whether that shared component was based only on edge geometry or also on additional visual cues.

Technical details regarding the machine‐learning algorithm, training strategy, cross‐validation procedure, annotation handling, and performance metrics are provided in the Supporting Information.

### 2.7 Statistical Analysis

Inter‐clinician agreement and model‐clinician agreement were quantified using Cohen's κ. For the unsupervised analysis, agreement between automatically identified groups and clinical annotations was assessed after optimal label matching. For the supervised analysis, model performance was evaluated using Cohen's κ and standard classification metrics, including accuracy, precision, recall, and F1‐score.

The performance of the geometry‐only and full‐feature supervised models was compared across repeated validation runs. Statistical significance was assessed using a two‐sample Student's *t*‐test. Full details of the validation strategy and complete performance metrics are reported in the Supporting Information.

## 3 Results

The distribution of wound edge annotations differed across the four expert clinicians, confirming variability in image‐based wound edge assessment (Table 1). The average inter‐clinician agreement was low, with a Cohen's κ of 0.20 ± 0.07.

**FIGURE 2:**
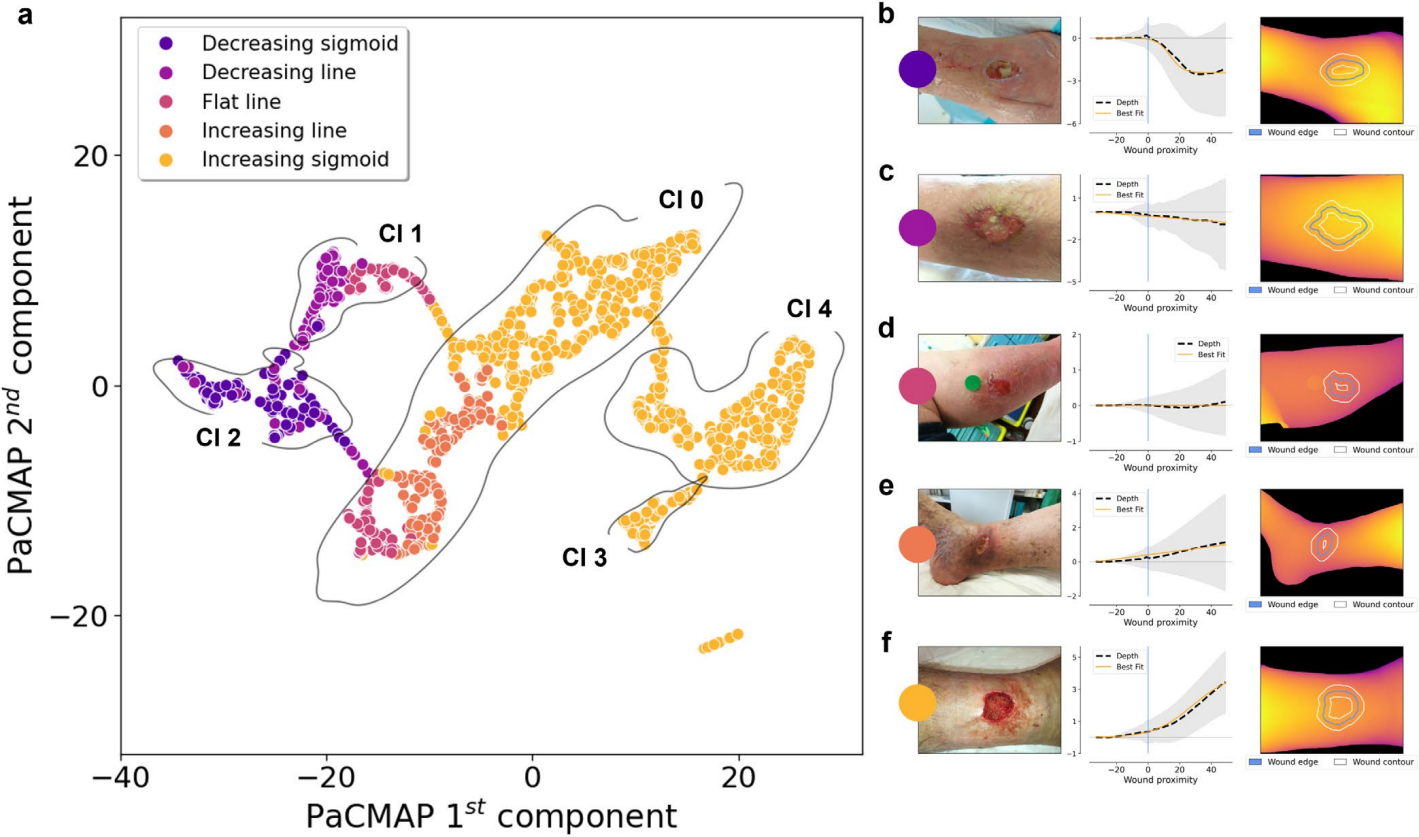
Unsupervised geometry‐based organisation of wound edge profiles. (a) Wound images were grouped according to the similarity of standardised depth‐derived edge profiles, without using clinical annotations during group generation; (b‐f) Representative examples of the automatically identified geometry‐based groups are shown together with the corresponding original image, edge‐depth profile, and estimated depth map.

In the unsupervised analysis, wound images were grouped using only standardised depth‐derived edge profiles, without using clinical annotations during group generation. The resulting geometry‐based groups are shown in (Figure 2). After post hoc comparison with clinicians' annotations, the correspondence between geometry‐based groups and clinical labels was minimal. The Adjusted Rand Index was 0.006, and the average Cohen's κ between unsupervised groups and clinical evaluations was 0.031 ± 0.006. These results show that depth‐derived wound edge geometry alone did not reproduce the clinical wound edge categories.

**FIGURE 3:**
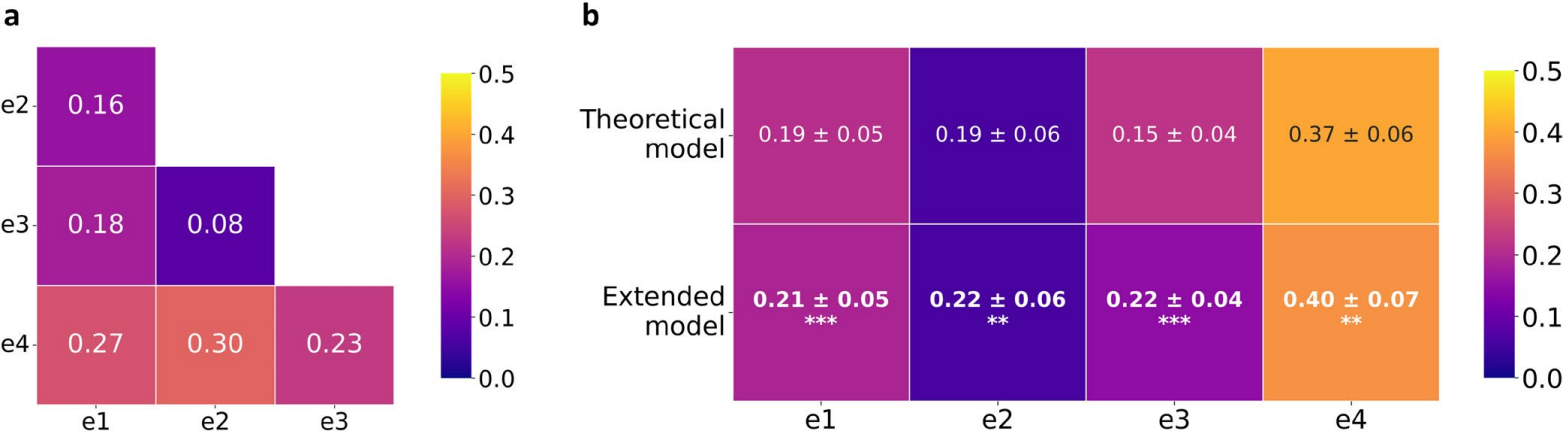
Agreement between expert clinicians and automated wound classifier. (a) Inter‐clinician Cohen's κ agreement is reported as the clinical benchmark for interpreting model performance. Statistical significance refers to the comparison between the two supervised models across repeated validation runs; (b) Cohen's κ agreement between individual expert clinicians and the supervised models trained using either geometry‐only descriptors or the full set of image‐derived descriptors. **(*p*‐value < 0.01), ***(*p*‐value < 0.001). Complete performance metrics and feature‐importance analyses are reported in the Supporting Information.

In the supervised analysis, clinical annotations were used to train models aimed at reproducing clinicians' wound edge classifications. Two models were compared: A geometry‐only model based on depth‐derived edge‐profile descriptors and a full‐feature model incorporating additional image‐derived descriptors. The geometry‐only model achieved an average Cohen's κ of 0.21 ± 0.03 when compared with individual clinicians. The full‐feature model achieved an average Cohen's κ of 0.24 ± 0.03, significantly higher than the geometry‐only model across repeated validation runs (Figure 3b), according to a two‐sample *t*‐test. For reference, inter‐clinician agreement is reported in Figure 3a. Complete supervised classification metrics, including accuracy, precision, recall, F1‐score, and feature‐importance analyses, are provided in the Supporting Information.

## 4 Discussion

The main finding of this study is that wound edge classification from clinical photographs cannot be fully explained by edge geometry alone. Although the geometry‐based analysis produced coherent and interpretable edge profiles, these profiles did not correspond closely to the labels assigned by expert clinicians. This suggests that current clinical wound edge assessment relies on a broader visual interpretation than the formal definition of edge morphology alone.

This result is clinically relevant because wound edge categories are commonly used as part of structured wound assessment tools, yet their interpretation remains subjective. The low inter‐clinician agreement observed in our dataset confirms that even expert assessment is affected by substantial variability, particularly when based on two‐dimensional images without tactile information, clinical context, or alternative viewing angles.

The improved performance of the classifier incorporating additional visual descriptors suggests that clinicians' rivacues may be clinically meaningful, but they are not always explicitly separated from edge morphology in standard scoring systems. Their influence may therefore contribute to variability in wound edge classification.

The proposed approach should not be interpreted as a replacement for clinicaljudgement, but as a potential decision‐support tool for standardising wound documentation, especially in teledermatology and longitudinal follow‐up. By providing reproducible quantitative descriptors, automated analysis may help reduce variability and improve comparability between clinicians, centres, and time points.

### 4.1 Strengths

A major strength of this study is the use of real‐world wound photographs acquired during routine clinical practice, reflecting the variability of image quality, anatomical sites, and acquisition conditions encountered in telemedicine and outpatient wound care. In addition, each image was independently evaluated by multiple expert clinicians, allowing model performance to be interpreted in relation to human inter‐observer variability rather than against an artificial single‐label ground truth. Finally, the proposed pipeline provides a standardised way to quantify wound edge morphology, supporting more reproducible wound documentation.

### 4.2 Limits

This study is limited by its monocentric retrospective design and by the use of routine clinical photographs, which may not fully capture the heterogeneity of wound types, imaging conditions, anatomical sites, and skin phototypes encountered in broader clinical practice. Multicentre validation in more diverse cohorts is therefore required.

In addition, the analysis relied on two‐dimensional images and AI‐derived segmentation and depth estimation, which may be affected by image quality, lighting, acquisition angle, anatomical curvature, and visual confounders. Finally, clinical annotations were used as the reference standard despite low inter‐clinician agreement, indicating the absence of an independent anatomical ground truth. The results should therefore be interpreted as a proof of concept requiring further external validation.

## 5 Conclusions

Wound edge classification remains one of the most subjective components of structured wound assessment. In this study, agreement among expert clinicians was low, confirming the difficulty of assigning reproducible edge categories from routine two‐dimensional wound photographs.

Geometry‐based analysis provided coherent and interpretable descriptions of the wound edge, but it did not reproduce clinical annotations. In contrast, a supervised model incorporating both edge geometry and additional visual descriptors achieved agreement comparable to inter‐clinician variability. These findings suggest that clinical wound edge assessment is not based on geometry alone, but also reflects broader visual cues such as wound shape, colour appearance, and surface pattern.

Automated image‐based analysis may therefore support more standardised and reproducible wound edge documentation, particularly in teledermatology and longitudinal monitoring. Future multicentre studies including diverse skin phototypes, standardised acquisition protocols, and external validation are needed before translation into clinical decision‐support workflows.

## Author Contributions

C.Z.: Data curation, investigation, methodology, validation, conceptualisation, and writing – review and editing. T.G.: Investigation, visualisation, methodology, formal analysis, and writing – original draft. Y.M.: Data curation, visualisation, validation, writing – review and editing. D.G.: Data curation, visualisation, validation, writing – review and editing. L.R.: Data curation, validation, writing – review and editing. A.N.: Data curation, validation, writing – review and editing. M.F.: Data curation, investigation, visualisation, formal analysis, and writing – original draft. G.C.: Supervision, resources, project administration and writing – review and editing. D.R.: Supervision, resources, project administration and writing – review and editing. M.L.P.: Supervision, resources, project administration and writing – review and editing. A.P.: Supervision, resources, project administration and writing – review and editing. All authors have read and approved the final manuscript.

## Ethics Statement

This study adhered to ethical guidelines for survey research. Given the study's observational nature and the anonymity of the responses, formal ethical committee approval was not required. Participants were informed about the study's objectives, the voluntary nature of participation, and data confidentiality. No personal or sensitive health data was collected.

## Conflicts of Interest

The authors declare no conflicts of interest.

## Supporting information


**Data S1:** This supplementary material provides further details regarding the study population and the methodological framework. Specifically, it includes a detailed description of the patients’ demographic and clinical characteristics, alongside comprehensive overview of the mathematical models, algorithms, and workflow used for clinical annotation, image analysis, and segmentation, in order to ensure methodological rigor and the reproducibility of the model development process. The statistical analyses performed are also reported and provide a detailed comparison of the model's performance metrics

## Data Availability

The data that support the findings of this study are available from the corresponding author upon reasonable request. The code developed for the network‐based features extraction and analysis is publicly available on GitHub at https://github.com/Nico‐Curti/Deepskin.
