# Supplementary material for: Toward Objective Wound Edge Classification in Clinical Practice
Source: Exp Dermatol. 2026 Jun 5;35(6):e70287. doi: 10.1111/exd.70287 (PMC13241752; doi:10.1111/exd.70287)
Supplement: Supplementary file 1 — Data S1: This supplementary material provides further details regarding the study population and the methodological framework. Specifically, it includes a detailed description of the patients’ demographic and clinical characteristics, alongside comprehensive overview of the mathematical models, algorithms, and workflow used for clinical annotation, image analysis, and segmentation, in order to ensure methodological rigor and the reproducibility of the model development process. The statistical analyses performed are also reported and provide a detailed comparison of the model's performance metrics [file EXD-35-e70287-s001.docx]

**Toward Objective Wound Edge Classification in Clinical Practice**

Corrado Zengarini ^1, 2, *^, Tommaso Giacometti ^3, 4, *^, Yuri Merli ^1, 2, †^, Davide Griffa ^1, 2, †^, Luca Rapparini ^1, 2^, Alessio Natale ^1, 2^, Michele Fruci ^3, 5^, Gastone Castellani ^1, 6^, Daniel Remondini ^3, 4^, Michelangelo La Placa^1, 2^, Alessandro Pileri ^1, 2^, Michela Starace ^1, 2, ‡^, Nico Curti ^3, 4, ‡^

^1^ Department of Medical and Surgical Sciences, University of Bologna, 40138 Bologna (Italy)

^2^ Dermatology Unit, IRCCS Azienda Ospedaliero-Universitaria di Bologna, 40138 Bologna (Italy)

^3^ Department of Physics and Astronomy, University of Bologna, 40127 Bologna (Italy)

^4^ INFN Bologna (Italy)

^5^ IRCCS Istituto delle Scienze Neurologiche di Bologna, Neuroscience Biobank of Bologna (BNB), 40139 Bologna (Italy)

^6^ IRCCS Azienda Ospedaliero Universitaria di Bologna, 40138 Bologna (Italy)

^*^ These authors contributed equally to this work

† *Corresponding Author:* D. Griffa ([davide.griffa2@unibo.it](mailto:davide.griffa2@unibo.it)), Y. Merli ([yuri.merli2@unibo.it](mailto:yuri.merli2@unibo.it))

‡ Both authors contributed equally to this work and share seniorship.

**Author Information**

- **Dr. Corrado Zengarini:** (0000-0002-3323-9216) [corrado.zengarini2@unibo.it](mailto:corrado.zengarini2@unibo.it)
- **Mr. Tommaso Giacometti:** (0009-0002-5734-4579) [tommaso.giacometti5@unibo.it](mailto:tommaso.giacometti5@unibo.it)
- **Dr. Yuri Merli:** (0000-0003-4959-5378) [yuri.merli2@unibo.it](mailto:yuri.merli2@unibo.it)
- **Dr. Davide Griffa:** (0009-0009-6822-8777) [davide.griffa2@unibo.it](mailto:davide.griffa2@unibo.it)
- **Dr. Luca Rapparini:** (0000-0002-4029-941X) [luca.rapparini2@studio.unibo.it](mailto:luca.rapparini2@studio.unibo.it)
- **Dr. Alessio Natale:** (0009-0001-3984-4708) [alessio.natale@studio.unibo.it](mailto:alessio.natale@studio.unibo.it)
- **Mr. Michele Fruci:** (0009-0008-9433-8091) [michele.fruci@ausl.bologna.it](mailto:m.fruci@ausl.bologna.it)
- **Prof. Gastone Castellani:** (0000-0003-4892-925X) [gastone.castellani@unibo.it](mailto:gastone.castellani@unibo.it)
- **Prof. Daniel Remondini:** (0000-0003-3185-7456) [daniel.remondini@unibo.it](mailto:daniel.remondini@unibo.it)
- **Prof. Dr. Michelangelo La Placa:** (0000-0002-6894-3350) [michelangelo.laplaca@unibo.it](mailto:michelangelo.laplaca@unibo.it)
- **Prof. Dr. Alessandro Pileri:** (0000-0002-1025-7171) [alessandro.pileri2@unibo.it](mailto:alessandro.pileri2@unibo.it)
- **Prof. Dr. Michela Starace:** (0000-0002-3981-1527) [michela.starace2@unibo.it](mailto:michela.starace2@unibo.it)
- **Dr. Nico Curti:** (0000-0001-5802-1195) [nico.curti2@unibo.it](mailto:nico.curti2@unibo.it)

**Supplementary Materials**

**Patient population**

The dataset was collected at a tertiary referral clinic for difficult-to-treat wounds within the Dermatology Unit of IRCCS Sant’Orsola-Malpighi University Hospital (Bologna, Italy), acting as a hub for complex cases referred from territorial outpatient services and first-level hospital wards, as well as for in-patients from the same hospital. Between March 2019 and October 2021, a total of 884 patients were evaluated at the tertiary “ALA” outpatient clinic for difficult-to-treat wounds within the Dermatology Unit of IRCCS Sant’Orsola-Malpighi University Hospital (Bologna, Italy). Over this period, 1,860 wound images were acquired during routine clinical practice and subsequently included in the present dataset. The cohort showed a slight female predominance and was largely composed of older adults, with more than four fifths of patients aged ≥60 years and a substantial fraction aged ≥80 years. Multimorbidity was highly prevalent, with most patients presenting at least one systemic comorbidity, most commonly cardiovascular disease, diabetes mellitus, reduced mobility, obesity and concomitant antithrombotic or immunomodulatory treatments. Lesions were predominantly chronic ulcers of venous or mixed arterial–venous origin involving the lower limbs, mainly located on the legs and feet, with an approximately balanced left–right distribution and a predominance of anterior and lateral surfaces. Compression therapy and debridement, most frequently with curette, were widely employed, and systemic antibiotics were prescribed in the great majority of cases, with vasoactive agents used in a minority. Patient group details are provided in **Sup. Table 1**.

**Sup. Table 1. Patient population and wound image dataset.** Demographic and clinical characteristics of the wound clinic cohort included in the study. Data are reported for 884 patients and for the corresponding 1,860 wound images, as appropriate.

† Percentages for comorbidity types refer to the proportion of patients with each condition; multiple comorbidities per patient may occur.

‡ Percentages for lesion and treatment categories are based on 1,860 images; minor rounding discrepancies may occur.

|  | Variables | | Categories and Values (N, %) |
| --- | --- | --- | --- |
| Demographics  (patients) | **Sex** | | - **Female:** 496 (56.1%) - **Male:** 388 (43.9%) |
|  | **Age** | - **0–20:** 4 (0.5%) - **20–40:** 34 (3.8%) - **40–60:** 136 (15.4%) | - **60–80:** 293 (33.1%) - **80–100**: 412 (46.6%) - **100–120:** 5 (0.6%) |
| Comorbidities  (per patient) | **Number** | - **0:** 19 (2.1%) - **1:** 621 (70.2%) - **2:** 142 (16.1%) - **3:** 57 (6.4%) | - **4:** 31 (3.5%) - **5:** 10 (1.1%) - **6:** 4 (0.5%) |
|  | **Type†** | - **Cardiovascular disease:** 249 (28.2%) - **Diabetes mellitus:** 136 (15.4%) - **Reduced mobility:** 95 (10.7%) - **Obesity:** 73 (8.3%) - **Oral anticoagulant therapy:** 64 (7.2%) - **Autoimmune/immunologic: disease** 55 (6.2%) - **Lymphedema:** 53 (6.0%) - **Chemotherapy:** 29 (3.3%) - **Neurological disease:** 29 (3.3%) | - **Vasculitis:** 24 (2.7%) - **Systemic immunosuppressive therapy:** 19 (2.1%) - **Lymphoproliferative malignancy:** 18 (2.0%) - **Chronic systemic corticosteroid therapy:** 15 (1.7%) - **Substance abuse:** 8 (0.9%) - **Erosive pustular dermatitis:** 7 (0.8%) - **Osteomyelitis:** 5 (0.6%) - **Radiotherapy:** 5 (0.6%) |
| Lesion characteristics  (per image) | **Etiology‡** | - **Venous:** 359 (19.3%) - **Post-traumatic:** 359 (19.3%) - **Mixed arterial–venous:** 277 (14.9%) - **Arterial:** 216 (11.6%) - **Pressure:** 182 (9.8%) - **Vasculitic:** 95 (5.1%) - **Post-surgical:** 69 (3.7%) - **Tumor-related:** 60 (3.2%) - **Pyoderma gangrenosum-related:** 56 (3.0%) | - **Lymphatic:** 45 (2.4%) - **Diabetic:** 32 (1.7%) - **Scleroderma-related:** 26 (1.4%) - **Actinic:** 15 (0.8%) - **Neurological:** 15 (0.8%) - **Rheumatoid arthritis–related:** 9 (0.5%) - **Allergic contact dermatitis–related:** 4 (0.2%) - **Unknown:** 39 (2.1%) |
|  | **Anatomical site‡** | - **Leg:** 1,276 (68.6%) - **Foot:** 353 (19.0%) - **Gluteal region:** 54 (2.9%) - **Digits:** 54 (2.9%) - **Thorax:** 32 (1.7%) - **Thigh:** 30 (1.6%) - **Abdomen:** 24 (1.3%) - **Back:** 15 (0.8%) | - **Arm:** 11 (0.6%) - **Forearm:** 9 (0.5%) - **Hand:** 7 (0.4%) - **Lumbar region:** 6 (0.3%) - **Neck:** 2 (0.1%) - **Genital region:** 2 (0.1%) - **Head:** 2 (0.1%) |
|  | **Laterality‡** | | - **Left:** 947 (50.9%) - **Right:** 913 (49.1%) |
|  | **Orientation‡** | - **Anterior:** 627 (33.7%) - **Lateral:** 512 (27.5%) | - **Medial:** 422 (22.7%) - **Posterior:** 299 (16.1%) |
| Treatment  (per image) | **Compression therapy‡** | | - **High-pressure bandage:** 286 (15.4%) - **Medium-pressure:** 662 (35.6%) - **Low-pressure:** 911 (49.0%) |
|  | **Debridement‡** | - **Curette:** 1,408 (75.7%) - **CO₂ laser:** 192 (10.3%) | - **Silver nitrate:** 30 (1.6%) - **No debridement:** 232 (12.5%) |
|  | **Systemic therapy‡** | | - **Systemic antibiotic:** 1,730 (93.0%) - **Sulodexide:** 87 (4.7%) - **Mesoglycan:** 43 (2.3%) |

**Clinical annotation handling and label harmonization**

All images were independently annotated by four expert clinicians using the local BWAT/PWAT-derived wound edge categories reported in **Table 1** of the main manuscript: indistinct, attached, not attached, rolled-under, hyperkeratotic, and fibrotic edges.

After expert consensus, the rolled-under and fibrotic categories were merged for all downstream analyses because of their substantial visual overlap and limited reproducibility when assessed on two-dimensional clinical photographs. The final analytical label set therefore included indistinct, attached, not attached, rolled-under/fibrotic, and hyperkeratotic edges.

No majority-vote clinical ground truth was created. Instead, model performance was evaluated separately against each clinician in order to preserve inter-observer variability as a clinically meaningful benchmark.

**Automated wound segmentation and depth-map estimation**

Wound segmentation was performed using *Deepskin*^1,2^, an automated wound image segmentation model previously developed and validated for clinical wound photographs. The model was used to identify the wound area and generate a binary wound mask separating the lesion from surrounding skin and background. When multiple connected components were detected, only the largest wound component was retained for subsequent analyses, in order to reduce the influence of secondary lesions, artifacts, or non-wound objects. Automated segmentation outputs were visually checked by expert clinicians before feature extraction.

Relative depth maps were estimated from the two-dimensional RGB photographs using *Depth-Anything-V2*^3^. The model provides monocular relative-depth estimates rather than metric three-dimensional measurements. Therefore, depth-derived descriptors were interpreted as relative surface-shape features of the wound edge transition, not as absolute wound depth measurements.

The wound mask and the corresponding relative-depth map were combined to characterize the transition between peri-wound skin and wound bed. This transition region was then geometrically standardized using the rectification procedure described below.

**Wound Rectification Algorithm**

The aim of the rectification procedure was to transform the irregular peri-wound border into a standardized rectangular representation. This step was required because wound contours are highly variable across images and because direct comparison of edge profiles along irregular masks would introduce biases related to wound size, shape, and acquisition perspective.

For each wound mask, a transition band was generated around the wound contour by applying morphological operations to the binary mask. Dilation was used to sample the peri-wound region outside the wound border, whereas erosion was used to sample the inner region toward the wound bed. The same spatial transformation was applied to both the RGB image and the corresponding relative-depth map, producing aligned rectified representations of the wound edge region.

In the rectified image, one axis represents the normalized position along the wound perimeter, whereas the other axis represents the signed distance from the wound border, from the outer peri-wound region toward the inner wound bed. This common spatial reference allowed depth, color, and texture profiles to be extracted consistently across wounds.


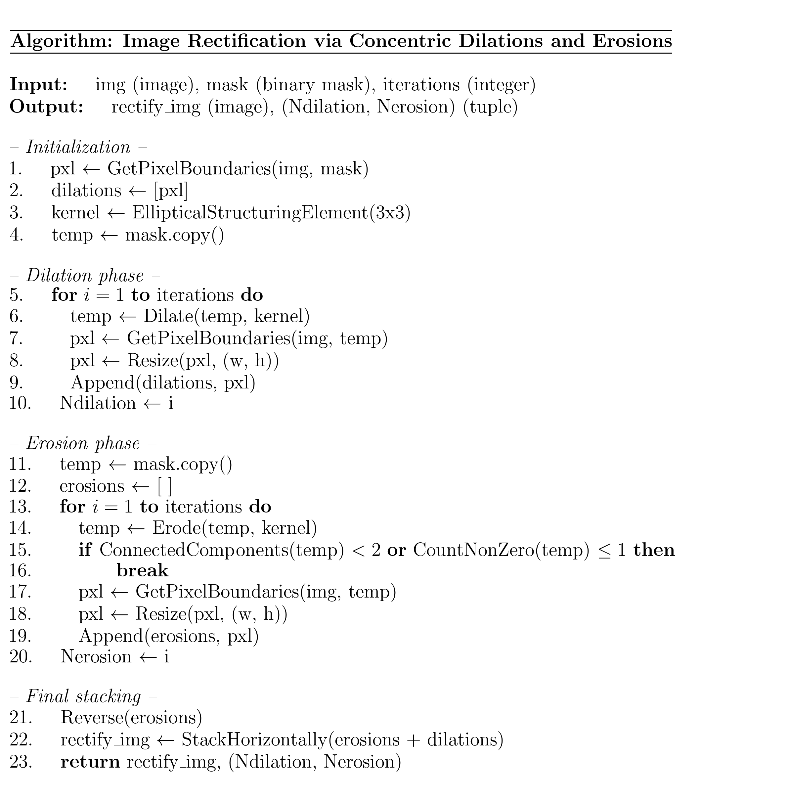


**Algorithm 1: Image rectification algorithm.** The pseudo code of the image rectification algorithm implemented for the analysis of wound edges. The same procedure was applied on both the RGB image and corresponding depth-map.

To reduce artifacts related to wound orientation and acquisition angle, the rectified depth region was corrected by fitting and removing a linear baseline before profile extraction.

**Depth-profile extraction and mathematical modelling**

For each rectified depth map, the relative-depth signal was summarized as a one-dimensional profile describing the transition from peri-wound skin to the wound bed. Profiles were obtained by averaging depth values along the normalized wound-contour axis, resulting in a depth trend as a function of distance from the wound border.

Each wound edge depth profile was fitted with two candidate functions: a linear model and a sigmoid model. The linear model was defined as:

$$f_{lin}\left( x \right)= ax+ b$$

where $a$ represents the slope and $b$ the intercept. The sigmoid model was defined as:

$$f_{sig}\left( x \right)= \frac{L}{\left( 1 +\exp\left( -k \left( x - x_{0} \right) \right) \right)}+ b$$

where $L$ represents the amplitude, $k$ the steepness, $x_{0}$ the inflection point, and $b$ the baseline offset.

To determine the most appropriate representation for each profile, the two fitted models were compared using the Akaike Information Criterion (AIC), which balances model goodness-of-fit with model complexity. Given the higher number of parameters in the sigmoid model, a penalty of three AIC points was applied to prevent overfitting and ensure a fair comparison. The best-fitting function was selected as the one yielding the lowest penalized AIC value. Additionally, profiles exhibiting an absolute linear slope below 0.05 were categorized as *“flat”*, representing negligible depth variation across the wound edge.

This systematic fitting and model selection procedure allowed the classification of wound edge profiles into distinct morphological trends, flat, linear, or sigmoidal, providing a compact and objective representation of edge geometry for subsequent analysis.

**Wound edge features**

The following table summarizes all the features extracted from the wound edge images. The unsupervised clustering model and the XGBoost model trained exclusively on edge profile information include only the features grouped under the category *“Edge profile”*, while the comprehensive model incorporates the full set of morphological, colorimetric, and textural descriptors.

**Sup. Table 2. Extracted wound-edge descriptors.** List of image-derived descriptors extracted from the rectified wound edge region and from the wound mask. The geometry-only analyses included only edge-profile descriptors, whereas the full-feature supervised model included edge-profile, morphological, colorimetric, and textural descriptors.

| Feature Type | **Feature name** | | **Description** |
| --- | --- | --- | --- |
| Edge profile | Mean of the profile | | Mean of the wound edge profile depth estimated via *Depth-Anything-v2* in function of the wound proximity (vector) |
|  | Standard deviation of profile | | Standard deviation of the wound edge profile depth estimated via *Depth-Anything-v2* in function of the wound proximity (vector) |
| Morphological features | Wound Area | | Area of the wound obtained from the *Deepskin* mask |
|  | Wound Perimeter | | Perimeter of the wound obtained from the *Deepskin* mask |
|  | Equivalent Perimeter | | $2\sqrt{\frac{A}{\pi}},$ where *A* is the wound area |
|  | Aspect Ratio | | $\frac{w}{h},$ where *w* and *h* are the width and height of the upright bounding box |
|  | Extent | | $\frac{A}{{BB}_{A}},$ where *A* is the wound area and ${BB}_{A}$ is the bounding box area |
|  | Circularity | | $4\pi\frac{A}{p^{2}},$ where *A* is the area and *p* the perimeter |
|  | Convex hull Area | | Area of the convex hull |
|  | Convex hull Perimeter | | Perimeter of the convex hull |
|  | Convexity | | $\frac{A}{{CH}_{A}}$, where *A* is the area and ${CH}_{A}$ is the convex hull area |
|  | Major Axis | | Major axis of the best fitting ellipse |
|  | Minor Axis | | Minor axis of the best fitting ellipse |
|  | Elongation | | Ratio between the wound area and the number of erosions required for the complete removal of the object |
|  | Circular Variance | | Mean square error with respect to the minimum enclosing circle of the wound |
|  | Elliptical Variance | | Mean square error with respect to the minimum enclosing ellipse of the wound |
|  | Sphericity | | $\frac{r_{in}}{r_{en}}$, ratio between maximum inscribed circle and minimum enclosing circle |
| Image moments | $m_{ij}$  ${mu}_{ij}$  ${nu}_{ij}$ | i, j ∈ {0, 1, 2, 3} | All image moments computed according to the OpenCV Moments implementation |
| Color features | RGB-equalized color profiles | | Profile means as a function of distance from the wound border of the R, G and B channels with a histogram equalization of the RGB domain |
|  | HSV-equalized color profiles | | Profile means in function of the wound proximity of the R, G and B channels with a histogram equalization of the HSV domain |
|  | LAB-equalized color profiles | | Profile means in function of the wound proximity of the R, G and B channels with a histogram equalization of the LAB domain |
| Haralick features^4^ | RGB equalization Haralick | | Haralick features for the grayscale converted image after RGB equalization |
|  | HSV equalization Haralick | | Haralick features for the grayscale converted image after HSV equalization |
|  | LAB equalization Haralick | | Haralick features for the grayscale converted image after LAB equalization |

**Unsupervised geometry-based analysis**

The unsupervised analysis was designed to test whether wound edge geometry alone could generate groups corresponding to clinical wound edge categories. Clinical annotations were not used during group generation. Only depth-derived edge-profile descriptors were included in this analysis.

Before clustering, edge-profile descriptors were standardized to reduce the influence of feature scale. A dimensionality-reduction step with PaCMAP^5^ was then applied to obtain a compact representation of profile similarity. Clustering was performed using HDBSCAN^6^, which was selected because it does not require the number of clusters to be specified a priori and can identify low-density observations as noise.

Because cluster identifiers are arbitrary and do not directly correspond to clinical labels, the comparison between unsupervised clusters and clinician annotations was performed only post hoc. Optimal cluster-to-label matching was obtained using the Hungarian algorithm^7^, maximizing the correspondence between automatically identified groups and clinical categories.

Agreement between unsupervised clusters and clinical annotations was quantified using the Adjusted Rand Index (ARI) and Cohen’s κ. Cohen’s κ was computed separately for each clinician after optimal label matching, and results were summarized as mean ± standard deviation across clinicians.

**Supervised classification analysis**

Supervised classification was performed to evaluate whether clinicians’ wound edge annotations could be reproduced from image-derived descriptors. In contrast to the unsupervised analysis, clinical labels were used during model training.

Two XGBoost classifiers^8^ were compared. The first model was trained using only depth-derived edge-profile descriptors and was used to test whether wound edge geometry alone was sufficient to reproduce clinical classifications. The second model was trained using the full feature set, including edge-profile, morphological, colorimetric, and textural descriptors.

The following XGBoost parameters were used: 200 (*n_estimators*), 6 (*max_depth*), 0.1 (*learning_rate*), 0.8 (*subsample*), 0.8 (*colsample_bytree*), “multi:softmax” (*objective*), “gbtree” (*booster*), 0 (*gamma*), 1 (*min_child_weight*), 0 (*reg_alpha*), 1 (*reg_lambda*), and “auto” (*tree_method*). Class imbalance was handled by no weighting.

Because each image was independently annotated by four clinicians, model performance was evaluated separately against each clinician rather than against a majority-vote label. This strategy avoided imposing an artificial single ground truth and allowed model performance to be interpreted in relation to inter-clinician variability.

Model performance was assessed across repeated validation runs. For each run, training and validation splits were generated according to the same procedure for the geometry-only and full-feature models. Performance was summarized using Cohen’s κ, accuracy, precision, recall, and F1-score.

**Statistical analysis and performance metrics**

Inter-clinician agreement, model-clinician agreement, and post hoc agreement between unsupervised clusters and clinical labels were quantified using Cohen’s κ. Cohen’s κ was used as the primary agreement metric because it accounts for chance agreement and is more informative than raw accuracy in the presence of class imbalance. For supervised multiclass classification, additional metrics included accuracy, precision, recall, and F1-score.

The geometry-only and full-feature supervised models were compared across repeated validation runs. Statistical significance was assessed using a two-sample Student’s t-test applied to the distribution of Cohen’s κ values obtained across repeated runs.

**Sup. Figure S1: Detailed supervised classifier outputs. a.** Distribution of Cohen’s κ agreement values between individual clinicians and the supervised automated classifier across validation runs; **b.** Feature-importance analysis of the full-feature XGBoost model, showing the ten highest-ranking descriptors contributing to wound edge classification. These analyses complement the main Figure 3, which focuses on the comparison between geometry-only and full-feature models and on inter-clinician agreement.


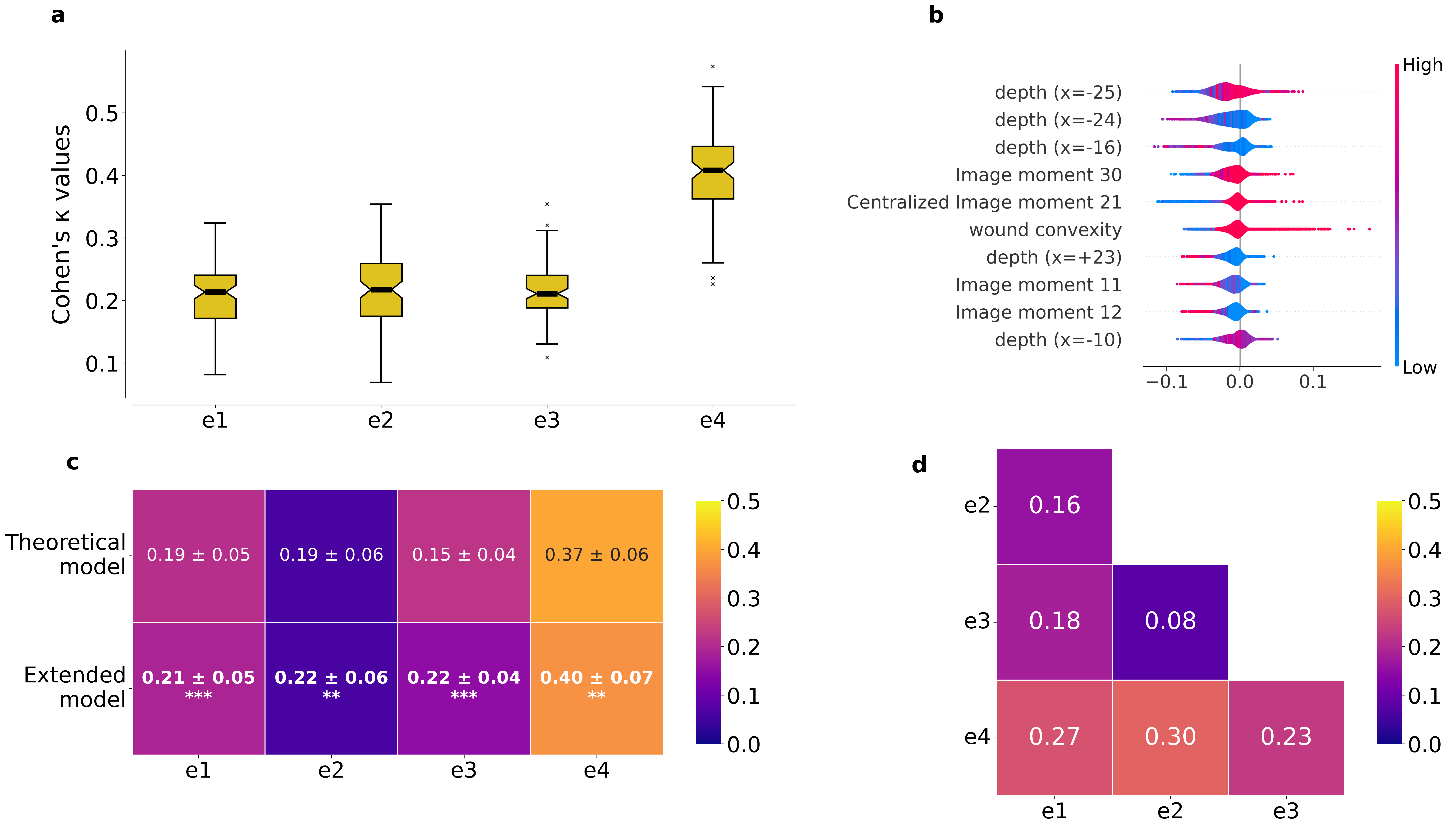


**Supervised model performance comparison**

**Supplementary Figure S2** reports the comparison of classification metrics between the geometry-only XGBoost model and the full-feature XGBoost model. Across repeated validation runs, the full-feature model showed higher performance than the geometry-only model for the evaluated metrics, supporting the hypothesis that clinical wound edge classification is influenced by visual descriptors beyond the local depth-derived edge profile.


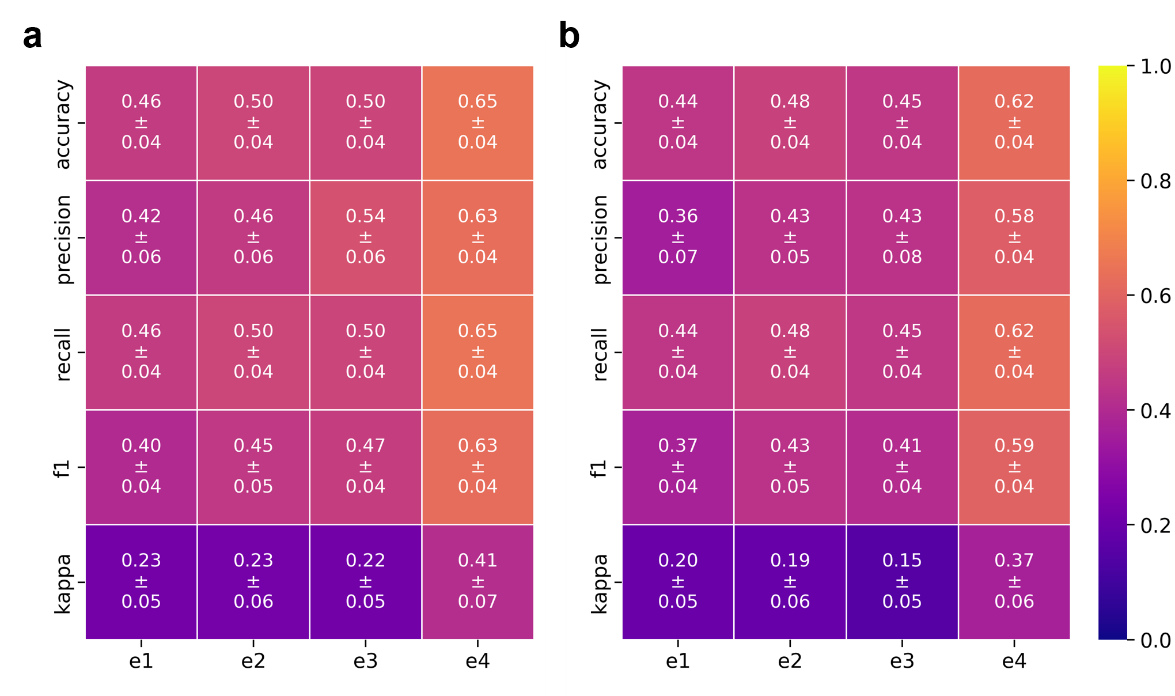


**Sup. Figure S2: XGBoost metrics.** All the metrics are substantially higher for the model trained with all the features; **a.** model trained with only the wound edge profile features; **b.** model trained with all the features extracted from the wound edge images.

**Feature importance for the profile-only XGBoost model**

**Supplementary Figure S3** shows the ten most influential features identified for the XGBoost model trained exclusively on depth-derived edge-profile descriptors. The highest-ranking predictors were located in regions immediately adjacent to the wound border, indicating that the local depth transition between peri-wound skin and wound bed contains reproducible geometric information.

However, despite the relevance of these local profile descriptors, the profile-only model achieved lower agreement with clinicians than the full-feature model. This finding supports the interpretation that edge-profile geometry contributes to clinical wound edge classification but does not fully explain it.


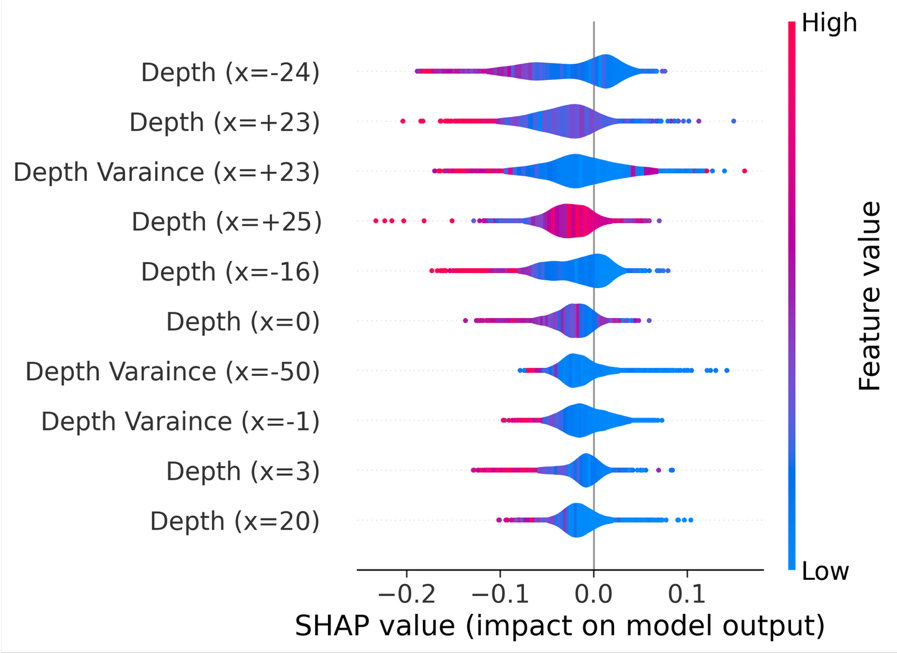


**Sup. Figure S3: SHAP values for the only profile XGBoost model.** Top 10 SHAP most important features extracted from the XGBoost model trained with only the wound edge profile information.

**Interpretation of top-ranking full-model features**

The most influential features in the full-feature XGBoost model included both local depth-profile descriptors and global morphological descriptors. The prominence of depth-profile values near the wound border supports the role of local geometric transitions in the visual assessment of wound edge morphology.

At the same time, the contribution of image moments and convexity indicates that clinicians may also rely on broader spatial information, including wound shape, contour regularity, asymmetry, and structural organization of the wound margin. These descriptors may visually correspond to irregular wound borders, hyperkeratotic thickening, localized accumulation of devitalized tissue, or residual material after local procedures.

Overall, the feature-importance analysis supports the main finding that clinical wound edge classification is not determined by edge geometry alone, but likely reflects the integration of local edge morphology with broader visual cues.

**Technical considerations and failure modes**

Depth-derived descriptors should be interpreted as relative image-based measures rather than as absolute anatomical depth measurements. Monocular depth estimation may be affected by image acquisition angle, illumination, shadows, reflective surfaces, wound dressing residues, tattoos, nail enamel, and other visual confounders. Similarly, segmentation quality directly affects all downstream morphological and edge-profile descriptors.

For this reason, the proposed pipeline should be considered a reproducible image-analysis framework for wound edge characterization, not a substitute for direct clinical or three-dimensional anatomical assessment. External validation in multicenter datasets including diverse skin phototypes and acquisition conditions remains necessary.

**References**

1. Curti N, Merli Y, Zengarini C, et al. Automated Prediction of Photographic Wound Assessment Tool in Chronic Wound Images. *J Med Syst*. 2024;48(1):14. doi:10.1007/s10916-023-02029-9

2. Curti N, Merli Y, Zengarini C, et al. Effectiveness of Semi-Supervised Active Learning in Automated Wound Image Segmentation. *IJMS*. 2022;24(1):706. doi:10.3390/ijms24010706

3. Yang L, Kang B, Huang Z, et al. Depth Anything V2. *arXiv*. Preprint posted online 2024. doi:10.48550/ARXIV.2406.09414

4. Haralick RM, Shanmugam K, Dinstein I. Textural Features for Image Classification. *IEEE Trans Syst, Man, Cybern*. 1973;SMC-3(6):610-621. doi:10.1109/TSMC.1973.4309314

5. Wang Y, Huang H, Rudin C, Shaposhnik Y. Understanding How Dimension Reduction Tools Work: An Empirical Approach to Deciphering t-SNE, UMAP, TriMAP, and PaCMAP for Data Visualization. Published online 2020. doi:10.48550/ARXIV.2012.04456

6. McInnes L, Healy J, Astels S. hdbscan: Hierarchical density based clustering. *JOSS*. 2017;2(11):205. doi:10.21105/joss.00205

7. Kuhn HW. The Hungarian method for the assignment problem. *Naval Research Logistics*. 1955;2(1-2):83-97. doi:10.1002/nav.3800020109

8. Chen T, Guestrin C. XGBoost: A Scalable Tree Boosting System. Published online 2016. doi:10.48550/ARXIV.1603.02754
